# Novelty, Stress, and Biological Roots in Human Market Behavior

**DOI:** 10.3390/bs4010053

**Published:** 2014-02-14

**Authors:** Alexey Sarapultsev, Petr Sarapultsev

**Affiliations:** Institute of Immunology and Physiology (IIP) of the Ural Division of Russian Academy of Sciences, 106 Pervomaiskaya str, Ekaterinburg 620219, Russia; E-Mail: p.sarapultsev@iip.uran.ru

**Keywords:** stress, novelty, behavior, crises, markets

## Abstract

Although studies examining the biological roots of human behavior have been conducted since the seminal work Kahneman and Tversky, crises and panics have not disappeared. The frequent occurrence of various types of crises has led some economists to the conviction that financial markets occasionally praise irrational judgments and that market crashes cannot be avoided a priori (Sornette 2009; Smith 2004). From a biological point of view, human behaviors are essentially the same during crises accompanied by stock market crashes and during bubble growth when share prices exceed historic highs. During those periods, most market participants see something new for themselves, and this inevitably induces a stress response in them with accompanying changes in their endocrine profiles and motivations. The result is quantitative and qualitative changes in behavior (Zhukov 2007). An underestimation of the role of novelty as a stressor is the primary shortcoming of current approaches for market research. When developing a mathematical market model, it is necessary to account for the biologically determined diphasisms of human behavior in everyday low-stress conditions and in response to stressors. This is the only type of approach that will enable forecasts of market dynamics and investor behaviors under normal conditions as well as during bubbles and panics.

## 1. Introduction

The reasons for financial market collapses have been examined for several decades; however, we remain unable to accurately predict such crises. The frequent occurrence of various types of crises has led some economists to the conviction that financial markets occasionally reward irrational judgments and that market crashes cannot be avoided a priori [1]. The source of this phenomenon is thought to be irrational human behavior when making decisions (e.g., risk seeking) [2]. It follows that irrational components in economic behavior almost automatically rule out the success of any economic forecasts. The opinion of John Marks Templeton, a highly reputable investor in the recent past, was that irrational character is accounted for by the peculiarities of the modern world: “…the most expensive words in the English language are, ‘This time it’s different’” [3]. However, a comparison of the behaviors of stock gamblers during stock crises in different time periods demonstrated that a gambler’s behavior does not depend on the time of or reason for the occurrence of the crisis [3,4,5,6].

## 2. Occam’s Razor

There exist sufficiently standard mechanisms determining the behavior of complex biological systems, human beings among them. This was proved by three ethologists—Konrad Lorenz, Nikolaas Tinbergen, and Karl von Frisch—who received the Nobel Prize in physiology and medicine for this discovery in 1973 [7,8]. K. Lorenz’s work (1965) emphasized that the basis of any research of human behavioral mechanisms must be the Occam’s razor principle, which when studying the behavior of animals possessing psyche, is described by Lloyd Morgan’s Canon: “in no case is an animal activity to be interpreted in terms of higher psychological processes if it can be fairly interpreted in terms of processes which stand lower in the scale of psychological evolution and development” [4,7,9,10]. It is important to note that the biological roots of human behavior, like those of other biological species, are innate, inherited, and cannot be eliminated or suppressed. However, the integral conduct is formed solely as a result of the superposition of acquired behavioral patterns on the innate ones [4]. 

In an analysis of human behavior, especially regarding financial decision-making, a Keynesian beauty contest might be used [11,12,13]. In his work, Keynes described a newspaper contest in which 100 photographs of faces were displayed. The winner would be the reader who selected six faces that were closest to the most popular selections of the combined lists of all readers. The best strategy, Keynes noted, was not to choose one’s personal favorites. It was to select the faces that others would think prettiest. Similarly, in speculative markets, Keynes indicated that an individual wins not by picking the soundest investment, but by picking the investment that others, who are playing the same game, will soon bid up higher [11,12,13]. However, this concept is not always applicable to the markets because it is time- and labor-intensive for the individual and requires the fulfillment of a chain of logical reasoning. Although this principle works well for laboratory conditions, the lack of time and the presence of novelty (which is very typical for markets) do not fit. Instead, stress occurs, and stressed animals (and people) primarily focus on fixed prerequisites (*i.e.*, a fixed set of actions). Novelty and consequent stress also may result in derived activity, manifested behaviorally as solving a task using the most simple and typical actions possible [14].

## 3. Physiology of Stress

When studying the mechanisms that form the basis of decision-making, especially financial decision-making, it is necessary to recognize that most decisions are made under stress [4] because, “Stress is the nonspecific body response to any demands presented to it” [15]. It is peculiar that the term “stress” was first introduced in physiology and psychology by Walter Cannon, who described the fight-or-flight response as a universal psychological response to a threat [16]. Here, the severity of stress depends on both the degree of the factor impact and the innate peculiarities of the body [17].

The main trigger activating the stress response is based in the brain [18,19,20,21]. In a number of works on neurophysiology, it was demonstrated that several brain divisions, including the amygdaloid complex (corpus amygdaloideum) [22], the anterior insula and the nucleus accumbens [19,20], are activated along with an irrational behavior. These researchers suggested that an excessive activation of certain brain divisions is responsible for the irrational behavior of investors, which is observed so frequently in real markets [18]. The key link in this chain of reactions is the release of corticotropin-releasing hormone (CRH) by the hypothalamus. The principal function of this hormone is to initiate adrenocorticotropic hormone synthesis by the hypophysis, which stimulates the secretion of glucocorticoids by the adrenal glands [4].

The stress response involves the coordinated activation of the hypothalamic–pituitary–adrenal (HPA) axis and the brain’s monoamine neurotransmitter systems [23,24]. When a stimulus evokes a stress response, both systems are activated by the same central mechanism. The result is the elevation of plasma corticosteroids and increased brain monoaminergic activity [23,25].

The disruption of key monoaminergic neurotransmitter systems may contribute to widespread cognitive dysfunction [26,27] and behavioral changes [28]. For example, rats in which dopamine transmission was blocked exhibited a behavioral shift towards choosing freely available lab chow over preferred food that was only obtainable by lever-pressing [29]. Experimental rats chose this low effort/low reward arm substantially more often than controls [30]. Low activities of monoamine oxidase A (MAO-A), an enzyme that degrades amine neurotransmitters (e.g., dopamine, norepinephrine, and serotonin), have been linked to aggressive behavior. In addition, genetic studies have indicated important connections among several genetic variants of this enzyme, environment, and aggression [31,32]. However, the predictive validity of MAO-A for aggression remains uncertain [33].

Special attention has been directed to the HPA axis’s regulation of the stress response [34]. The release of HPA hormones into the blood positively correlates with the intensity of stressful situations; therefore, these hormones are well-suited to reflect differences among subjects regarding the extents of emotional activation [34]. Moreover, glucocorticoids (cortisol in humans and most mammals; corticosterone in rats and mice), the final output hormones of the HPA axis, have been implicated in a wide range of pathophysiological and psychopathological processes, including cardiovascular disease, immune suppression, altered gastrointestinal function, anxiety disorders, depression, and predisposition to drug self-administration [34,35,36].

## 4. Biological Roots of Human Behavior

Because the stress response of the endocrine system is in accordance with the nervous system’s reaction to exposure (including market event exposures) [37,38], and because hormone measurement techniques are routine, most researchers who have studied the influence of stress on stock gamblers have evaluated the correlation between endocrine system components (e.g., circulating hormone levels) and a specific human behavior involved in the stock exchange. While interpreting the results of the below-mentioned works, one should consider their limitations. Most of the reported conclusions have been described in terms of Pearson correlations [18,39], which may be based on several other factors (e.g., anxiety, motivation, health) and could lead to misinterpretations.

### 4.1. Cortisol Levels

A wide variety of natural stressors are known to elicit a corticosterone (cortisol in humans) response [40,41,42]. However, the degree of the elicited cortisol response varies widely across tasks [43]. Motivated performance tasks elicited cortisol responses if they were uncontrollable or characterized by a social-evaluative threat (*i.e.*, task performance could be negatively judged by others). Tasks containing both uncontrollable and social-evaluative elements were associated with the largest cortisol and adrenocorticotropin hormone changes and the longest times to recovery [43]. These findings are consistent with animal studies regarding the physiological effects of uncontrollable social threats, and they contradict the notion that cortisol is responsive to all types of stressors [43]. This may explain discrepancies among investigations of cortisol dynamics. For example, Coates and Herbert (2008) reported no correlations between cortisol levels and sales performance, but a correlation was detected (*r* = 0.48, *p* = 0.004) between the level of cortisol and the volatility of trader performance, representing a risk level in the use of a financial instrument for a set period of time [18]. This finding is explained by the fact that an increase in cortisol occurs as a response to an encounter with new and unexpected challenges [44].

A more prominent correlation was detected by the researchers [18] between the indeterminacy indicator at financial markets (the mode by which values of volatility for options to index were chosen) and the average daily level of cortisol (*r* = 0.86, *F* = 38.1, *p* = 0.001). It is difficult to summarize the psychological effect of glucocorticoids because their influence depends on the time of exposure. During a spike rise in the level of glucocorticoids, there occurs euphoria, increased motivation, and focused attention. In contrast, during a continuous rise in the level of glucocorticoids acting through the amygdaloid complex and hypothalamus, a selective shift in attention occurs that causes the individual to focus on negative expectations [45], experience soaring anxiety, and anticipate danger and risk even where there are none [38]. Therefore, it is very probable that a continuous rise in the level of glucocorticoids is capable of causing less risky behaviors among traders [18]. The optimal level of circulating glucocorticoids that enables adequate decision-making in an individual is, most probably, the low level characteristic of optimal physiology, as demonstrated by experiments on monkeys [46]. Among baboons, a low basal level of cortisol was detected only in silverbacks possessing at least one of the following features: they clearly perceived the neutral and threatening actions of their rival and responded differently to different actions. If the rival was an actual threat, they controlled the situation by starting a fight. Having experienced victory, their conduct was different than when they were defeated. In the latter case, they expelled frustration by displaced aggression (*i.e.*, on an unassociated “third party” [46]. Importantly, there exists a contrary opinion according to which a low level of cortisol is a marker of an increasing inclination to take risks and a fearless attitude toward losses. By this model, under stressful conditions that induce a rise in the level of cortisol, the inclination to take risks must decrease [47].

### 4.2. Plasma Testosterone Levels

Several studies have attempted to identify a relationship between the level of testosterone and the conduct of stock gamblers. It appeared that the testosterone index correlated drastically less well than cortisol with the level of volatility (*r* = 0.36, *F* = 3.1, *p* = 0.013), and testosterone levels among traders were higher when they won [18]. The existence of correlations between elevated testosterone and profit was discussed by the authors who referenced previous reports that testosterone increases search activity [48], willingness to take risks [49], and fearlessness in front of novelty [50,51]. It is probable that testosterone can induce the financial version of the “winner effect:” that previous wins increase its surge and intensify the drive for risk in the future because testosterone and its metabolites increase the surge of dopamine in brain regions responsible for risky behavior [19,52,53].

Other reports have renounced the generally accepted hypothesis concerning the intensification of the drive for risk under the influence of testosterone because it appeared that even direct introduction of testosterone resulted in fairer conduct at deals, rather than more aggressive behavior [54]. This finding was confirmed by N. Zethraeus who did not detect any statistically significant changes in the behavior of 200 women when estrogens or testosterone were introduced [55]. These authors attribute the contradiction between their results and previous data to the fact that the earlier studies examined salivary hormones using methods that were not sufficiently sensitive and potentially invalid [55]. Thus, the level of cortisol presumably must increase during periods of financial market collapse, and the level of testosterone must increase during periods of bubble formation [18].

### 4.3. Hereditary Factors Affecting Testosterone Levels

Following the publication of statistics concerning the influence of testosterone level on human behavior, reports emerged that attempted to identify hereditary factors that predicted changes in this hormone [39,56,57,58,59,60]. One study found that an increased level of androgens during fetal development is manifested phenotypically as an altered second-to-fourth digit length ratio (2D:4D) [61]. This represents a clear predictor of perinatal androgenization that can be used as a marker of the level of reproductive hormones in postnatal life [61]. In women, a low 2D:4D ratio is a predictor of success in sports [56,57], and it correlates not only with achievements in their field (in particular, fencing), but also with aggressive behavior during matches [59].

Investigators found it more difficult to compare heritable indicators of reproductive hormone levels with the peculiarities of financial market perceptions. Drawing from a study of the City of London traders, it was purported that altered digit length ratios predict the professional suitability of traders [39]. Yet, some researchers detected an association between elevated testosterone among women and a higher degree of risk aversion [58]. Other researchers, failing to detect any valid correlations among women, described a correlation between smaller 2D:4D values among men and an inclination to take financial risks [62].

## 5. The Role of Stress in Decision-Making

When comparing hormone levels and human behaviors in financial markets, it should be recognized that decisions, especially financial ones, are mostly made under stressful conditions [3,6]. Although many researchers have studied stress using laboratory animals [63,64,65], their works contribute significantly to the understanding of the mechanisms of decision-making under stress. According to several scholars [66], interspecies differences are not large enough to exclude extrapolations of correlations from extensively studied animal species to humans, for whom experimental data are scarce. For example, the prefrontal cortex as a whole shows considerable variation across species and differs markedly in the amounts of granular versus agranular cortex. However, similarities in the positions and connections of orbital and medial areas indicate that the orbital and medial prefrontal cortex is relatively comparable across species [66,67,68].

As demonstrated in rats exposed to a cold stressor, stressful conditions increase the concentration of corticosterone and testosterone relative to non-stressed animals [69]. Moreover, changes in the concentration of cortisol can determine the behavior of a stock gambler in the absence of stress, when a low level of cortisol is the marker of an increased inclination toward risk-taking and a fearless attitude toward loss. Stress, in turn, increases cortisol and lessens the inclination to take risks [47]. Most important in studying traders’ behaviors in the stock market, however, is their psychological reaction to acute situations, especially those leading to distress, which can drastically change standard human behavior.

The cross-talk between stress and cognition has been reviewed in detail elsewhere [70,71,72,73]. Opinions concerning the effects of stress on cognitive capabilities and decision-making are very diverse. Whereas some scholars [10] assert that stress does not influence cognitive performance, others propose that stress significantly shifts an individual’s attitude toward risks and upsets neuromechanisms involved in rational decision-making [18,74]. This divergence in study results may be explained by several factors. Firstly, nearly all regular bodily functions detected at rest change under stress. When introduced into an animal, many hormones (e.g., vasopressin, estrogens) increase motion activity if the animal is in a familiar cage, but decrease activity if the animal is in new or threatening surroundings. Also, the level of hormones (in particular, corticosterone) under stressful conditions depends on the basal blood pressure. At rest, a higher level of corticosterone is detected with rats exhibiting low blood pressure. Under stress, a higher level of corticosterone is detected with rats displaying higher blood pressure [4].

Secondly, a potential experimental error is a misconception regarding the degree to which stress impacts an individual. A study employing the classical Morris maze test of memory duration, in which a swimming rat can blunder upon an underwater islet only incidentally, indicated memory impairment in animals subjected to an increase in exogenic or endogenic corticosterone 30 min before the test [63]. However, this experiment did not address the fact that laboratory rodents originate from Norway rats (Rattus norvegicus), a semi-aquatic species that loves to bathe. Hence, two competing motivations are at play when rats are put into the pool: scrambling away from danger upon finding the islet and a low level of anxiety due to the comfort of being in the water [4].

## 6. Personal Differences

When studying behavior under stress, it also is necessary to account for the initial elevated nervous activity of the test subjects. In an experiment with dogs in which the possibility of having meat in a trough was 20%, whereas crackers were given to the dogs with a probability of 100%, cholerics (emotionally unstable extraverts) and phlegmatics (emotionally stable introverts) preferred crackers, that is, 100% refreshment. In contrast, melancholics (emotionally unstable introverts) and sanguines (emotionally stable extraverts) preferred meat, or 20% refreshment. A short annoying influence that increased the level of anxiety induced cholerics and phlegmatics to prefer meat. That is, after a disturbing influence, all animals chose the lower probability but higher quality refreshment [64]. This study exemplified the different roles of anxiety in the behavior of animals of different psychological types.

Similar data also were reported among people who, notwithstanding temperament type, showed significant differences in their sensitivities to anti-anxiety medicines. When comparing the sensitivities of people of different psychological types [75] to anti-anxiety medicines, introverts showing a moderate level of emotional stability were most tolerant to their effects. Extraverts showing a high level of neuroticism (*i.e.*, with a low level of emotional stability) experienced the strongest effects of the medication. Other psychological types, including neurotic introverts and emotionally stable extraverts, demonstrated approximately similar sensitivities to pharmacological intervention [4].

The results of the above animal studies [64,65] correlate with results reported by Porcelli and Delgado (2009), who studied human behavior under stress [74]. Those studies confirmed the concept that increasingly risky behavior is observed in people under stress and demonstrated that people under stress rely more on lower-level automatized systems in decision-making. This manifests in lower performance at memory tests and extended durations for making decisions after stress stimulation [74]. This affects decision-making behavior and differentially inhibits the operation of analytical reasoning processes, occasionally leading to incorrect decisions [74,76].

## 7. The Role of Motivation

The alteration of human behavior under stress is associated with positive and negative aspects as well as dominant motivations. As an example, consider a simple test that measures an individual’s ability to extrapolate by requiring the subject to follow an object hidden under one of several paper cones moving according to a certain pattern. Children demonstrate superior abilities to adults in this test. Adults see this activity as mere entertainment and are less interested in praise from the test conductor or in the candy hidden under the cone [4]. Interpretation of study results also can be influenced by the “reflection effect,” in which people perform more risky actions in case of loss, but they are not ready to take risks in case of winning. The reflection effect significantly increases after stress stimulation [74]. According to [77], while people normally are inclined to take risks in case of winning, after stress stimulation, they become inclined to accept increased risk, both at large and after bearing severe loss. In the author’s opinion, this reaction is explained by the fact that an increasing level of cortisol under stress causes increases dopamine, which is associated with a sense of euphoria and a desire for new sensations [77]. These empirical findings fully correlate with theoretical discussions by Kahneman and Tversky (1979), who established that individuals are risk-averse when faced with positive prospects and are risk-seeking when faced with negative prospects [2].

## 8. Wins and Losses

When studying the behavior of stock gamblers, one must examine the behavior of players during wins and losses, as there is a difference in the mechanism of brain functioning between expecting and receiving cash prizes [60]. Counterfactuals and regret have been studied behaviorally and experimentally, using functional magnetic resonance imaging [19,78]. Results indicate that motivations to obtain a reward and avoid a loss are realized through different brain mechanisms [79,80]. The anticipation of cash prizes activates a functional module that involves the interaction of zones of the limbic system, frontal lobes, and the striatum, a set of nodes in the subcortical basal region that undergo enhanced activation and play a central role in this process [46]. In these studies, the striatum under-reacted to prizes already received, whereas strong activation was detected in the frontal lobes [81]. The results of Lohrenz and others added to evidence suggesting a special role for the caudate regarding decision-making in trial-and-error tasks involving a diverse range of rewards, even those associated with social exchange [81,82].

These scientific data are extremely interesting, but the results of their practical applications are poor, and they are not likely to reflect the target audience. For a professional financial expert, the two states of profit attainment and suffering losses are common and expected and would not be expected to cause behavioral changes. Supporting this, a report by Lo and Repin (2002) demonstrated that experienced traders showed more subtle vegetative components and less powerful emotional reactions to news releases than did novices and that news releases presented no stress to the experienced traders [83].

Ongoing research continues to introduce novel ideas that can be translated into practice. For instance, the fictive error signal, which drives choice behavior and has a neural correlate in the ventral caudate, has been defined recently [82]. It is expected that reports situating fictive error signals within the framework of machine learning models will provide additional insight into normal human behavior and diseases of decision-making [82].

## 9. Gender-Specific Behavior

Gender-specific behaviors in response to stress also should be considered [56,84]. Among men, the stress reaction is characterized by obvious appetites toward risk [50]. Among women, insignificant stress responses increase appetites toward risk, whereas severe stress reactions dramatically decrease them [50]. The responses of the HPA axis and brain monoamine neurotransmitter systems show marked and consistent differences according to sex. Among women, differences exist according to the phase of the menstrual cycle, menopausal status, and pregnancy [85]. These gender-related differences in autonomic function may be the result of estrogen exposure, which attenuates sympathoadrenal responsiveness. This mechanism exists to protect the fetus from the adverse effects of maternal stress responses, especially excessive glucocorticoid exposure [85]. Thus, between puberty and menopause, adult women usually exhibit lower HPA axis and autonomic responses than men of the same age. This difference substantially alters their reaction to stressors [85]. Notably, there exists a point of view according to which gender socialization plays an equally important role in the formation of differences in stress responses [86].

## 10. Stress and Community

The stress situation is capable of changing behaviors not only of individuals, but also of communities. A social structure can be observed among animals existing in stable conditions, and this structure can be changed by chronic stress. For example, under conditions of food shortage, animals at upper levels in the hierarchy begin to be displaced by other animals. Among cloven-hoofed mammals that usually follow one of the mature stags, a threat (e.g., predators, fire) stimulates the reassignment of leadership functions to one of the older does [4]. In this respect, the conclusions of Coates and Herbert (2009) are relevant [18]. These researchers hypothesized that behavioral changes are caused by hormones with a minority of stock market participants influencing the behaviors of the others [18,87].

## 11. Conclusions

According to Selye (1976), stress is “the state manifested by the specific syndrome, which consists of all the non-specific induced changes in a biological system” [15]. A stressor may be a physical insult, such as trauma or injury, or physical exertion, particularly when the body is being forced to operate beyond its capacity. Other physical stressors include exposure to a novel environment, noise, overcrowding, and excessive heat or cold [88]. Exposure to a novel environment elicits anxiety-driven defensive responses [89]. Given this concept, a qualitatively different and novel type of behavior can occur only under stress [4], that is, as a reaction to an entirely new stimulant [65,90].

For the financial sector, these new stimulants take the form of crises and bubbles in the market. From a biological point of view, human behavior essentially is the same during crises accompanied by stock market crashes and during bubble growth when share prices exceed historic highs. During these periods, most market participants see something new for themselves, and this inevitably induces a stress reaction in them [4]. As a result, their endocrine profiles [84] and motivations change, leading to quantitative and qualitative differences in behavior [4].

From a biological point of view, human behavior essentially is the same during the first phase of crises, accompanied by stock market crashes, and during bubble growth, when share prices exceed historic highs. During these initial periods, most market participants see something new for themselves, and this inevitably induces a stress response in them [4]. Their endocrine profiles [84] and motivations consequently are affected, leading to appropriate quantitative and qualitative changes in behavior [4]. These changes, in turn, depend on the profit or loss the market movement has brought to them [65,79].

The role of novelty as an initiating factor of market bubbles was emphasized by Smith (2004), who asserted that novelty occurs only in the beginning of the growth period [3]. Subsequent crises are regarded as a follow-up and “pay-off.” However, a slump in prices for assets also is novel for a subset of market participants. At the early stages of bubble development, when an investor faces novelty, and at the stage of its “blow-off,” when investors grow anxious, innate mechanisms aimed at attaining a high-quality “refreshment” take predominance, even if chances of obtaining it are low. This exemplifies what psychologists call a “risky behavior” [2,4].

This may be the reason that all attempts of mathematicians to create models of financial markets ultimately fail. This fact is exemplified by the Long-Term Capital Management (LTCM) fund [91], whose sales strategies proved inefficient in the face of novelty even though members of the fund’s Board of Directors, Myron S. Scholes and Robert C. Merton, were awarded the Nobel Prize in Economic Sciences in 1997 for a new method to determine the value of derivatives [92,93,94]. Although LTCM managers and many other scientists and traders have created coherent and adequate models allowing effective management of investments over time, all of the models reflected, with some degree of conformity, investors’ behaviors (and, correspondingly, predicted asset price movements) during a particularly tranquil market. As soon as the market changed radically, owing to various reasons and occurring either in the beginning or the end of the market cycle, the novelty effect caused the model to no longer reflect reality.

We can conclude that practically all studies conducted to date are disadvantaged in terms of research methodology [18,74], research object selection [47], and/or the interpretation of results [18,87]. Indeed, despite all studies of the biological roots of human behavior conducted during the past four decades since the seminal work of Kahneman and Tversky [2], crises and panics have not disappeared. Instead, they occur now even more frequently. When developing a mathematical market model, it is necessary to account for the biologically determined diphasisms of human behavior, both under everyday low-stress conditions and under stress. This approach alone can enable forecasts of market dynamics and investor behavior during normal conditions as well as during bubbles and panics. Novelty indicators may be volatility indices (e.g., of shares or derivative products) or moments of inconsistency in the contemporary financial model (e.g., periods of abnormal pricing at options). This approach may refute Sornette’s postulate, which states that it is impossible to predict the moment when a crisis begins [1].

To solve this task methodologically, all studies must be divided at least into three groups. The first group must include studies implementing the search of genetically determined human qualities predisposing individuals to certain types of activities (here, financial activity). The second group must comprise studies investigating human behavior under low-stress situations. Results of such studies can be applied to market analysis, but only if the markets are tranquil and of streamline motion, the so-called “flat market”. The third group must list studies analyzing human behavior under simulated stressful conditions or collect information for an analysis conducted during real-life stress situations occurring in the stock market.

The most appropriate design for these three groups of studies, particularly for the third group, should include the following components: (1) Separation of experimental groups by gender, because gender strongly affects all of the characteristics and manifestation rates of behavioral responses [56,84,85]; (2) Determination of the basic characteristics of each individual using as daily indicators the urine [95] or plasma [96] levels of catecholamines, testosterone, and cortisol. This is because basal levels [58,62] and profiles of circadian excretion [97] of hormones can influence behavior in animals [98,99] and humans [100]; (3) Determination of patterns of increased nervous activity (e.g., that associated with type A behavior) that correlate with the nature of the individual’s reaction [101,102,103,104]; (4) Determination of the occurrence of a stress reaction at the moment of study. This fourth component is particularly important for human studies and may be accomplished using a survey [105] or by measuring blood leukocyte counts [35]. A second survey in the final phase of the experiment can serve as an indirect confirmation of stress during the study. The recently introduced Stress Overload Scale (SOS) may be used to detect short-term fluctuations in stress levels as an alternative to objective measures, which only reflect shifts in external circumstances [105].

Perhaps, it is the combination of those approaches that will help economists and mathematicians to develop and implement new models that will allow market participants and regulators to control their risks more precisely, which is the goal of any market actor.
